# The Contact Point and the Interproximal Space

**Published:** 1913-04-15

**Authors:** J. V. Conzett

**Affiliations:** Dubuque, Iowa


### THE CONTACT POINT AND THE INTERPROXIMAL SPACE.

BY J. V. CONZETT, D.D.S., DUBUQUE, IOWA.

In the making of a filling of any kind for the restoration of tooth tissue that has been lost by the ravages of caries, one of the most important considerations (and the one that is most frequently disregarded) is the restoration of the mesio-distal diameter of the tooth, which means the restoration of its normal contour, and the bringing of the tooth into its proper occlusal aspect as it pertains to the opposing teeth upon the other jaw.

Decay upon the approximal surface of the tooth begins just gingivally to the contact point, and as soon as the decay makes any headway a portion of the contact point is lost. As the decay progresses, more of the same portion of the tooth is lost, and as the tooth disappears and the contact flattens the tooth moves forward and the mesic-distal diameter of the tooth is shortened. If the decay is of slow progress and is neglected for a long period of time this loss is of considerable proportions and of very much importance, for the flattening of the contacts causes a strangulation of the septal tissue and a consequent loss of the same by the gingival portions of the approximating teeth moving closer, thus destroying the interproximal space and the contained tissue. The flattened contacts are not well adapted to keeping the food from crowding into the interproximal spaces, and the next step is a gingival irritation from the packing of fibrous foods into the unprotected spaces. This continued irritation is not only extremely uncomfortable, and sometimes very painful, but causes an inflammation that in time develops into a pyorrhea and the ultimate loss of the teeth involved.

This condition not only prevails with the presence of decay and in the unrepaired condition of the tooth, but if the filling is not properly made, if the contour of the tooth is not fully reproduced, the contact not properly placed and shaped, and the interproximal space thereby restored to its original shape and size, the same conditions of discomfort and gingival disease will prevail.

It will thus be seen that the restoration of the contour, the making of a proper contact point and the preservation or restoration of the interproximal space, are of the utmost importance, both from the standpoint of the comfort of the patient and the preservation of the tooth.

When a tooth presents with the decay in such a state that a portion of the proximal surface has been lost the first thing to do is to obtain sufficient space to properly restore the lost tissue. This is done by obtaining separation, and may be done in one of several ways. If the loss is not great it may be obtained by the tying of a loop of cotton twine between the teeth and knotting the ends together in such a way that the knot is drawn in between the teeth, and by increasing the number of knots a larger separation may be secured. This string swells by the absorption of the fluids of the mouth, and by swelling gradually causes the teeth to separate. It is quickly and painlessly done. Never use rubber for separating purposes, for it causes extreme pain and is likely to slip under the gingival margin and cause much damage to the septal tissue. I have seen great masses of tissue thus lost and a pocket made that was extremely hard to eradicate.

If a great deal of the tooth is lost by reason of decay, and there has been considerable falling together of the teeth, it may require considerable time and patience to properly reproduce the required space for full restoration. But it can be done. If time is of no particular importance the best way is to fill the cavity overfull with baseplate gutta-percha and allow the patient to masticate upon it for a time, when it will be found that some space has been obtained. If not enough, more gutta-percha may be placed in the same way and the process repeated until a sufficient space has been gained. If it is desired to more expeditiously obtain the space a mechanical separator is placed between the teeth, and as much separation obtained as possible at that time; then fill with gutta-percha to hold that which has been obtained and dismiss for a future sitting, when more may be gained in the same way if necessary. This may be repeated until the space is satisfactory.

The tooth, if normally situated and in normal approximal contact with the neighboring tooth, needs but to be brought to a normal contour, for its natural shape will be the proper shape to bring it into proper contact with its approximating tooth, but if the normal contact for any reason has been broad and flat, it will be best to correct the condition by making the contact small and round. For a broad contact invites the impaction of food between the teeth, and all such conditions must be avoided. Dr. Black illustrates the ideal contact by saying that it should be such as would be made by two marbles knuckling together. Both being rounded surfaces there would be the least possible amount of tissue in immediate contact, and yet the contact would be such that all of the surfaces of the tooth would be sloping toward the contact point, and the approximal embrasures would be best shaped to allow for the cleansing of the surfaces of the tooth and to prevent food from crowding into the interproximal space.

A contact so made will in the best possible manner make for clean margins of the filling. A contact that is broad will require a great deal more cutting of the tooth in the preparation of the cavity for filling in order to bring the margins of the completed filling into areas of immunity than would be required if the contact is made small and round. And if the contact is made a little larger in its mesio-distal diameter—that is, if a plus contact is made—there will be less need of bucco-lingual extension than if the contact is not so made.

The contact point should be placed slightly buccally to the median line of the tooth in order to be normally situated, providing the teeth are in normal approximal relation; and if not, then the judgment of the operator must decide where it will be of the greatest advantage to the patient and of the greatest good to the denture.

As I have said, the contact point must be so made that all of the lines of the filling slope toward it; that will mean that it cannot be placed upon the proximo-occlusal angle, as I have heard advised, for if it were so placed all of the lines of the filling could not slope toward it. But it should be placed very close to the occlusal surface, and then the marginal ridge being normally rounded produces the slight slope from the occlusal surface nearest approaching the ideal.

Even though a tooth have no cavity, if the contact has been broadened and flattened by the abrasive action of long years of mastication, as is very frequently the case in people of advanced years, it is good practice to separate the teeth, cut a cavity or cavities in one or both of the approximating teeth, as necessity may dictate, and properly restore contour, contact and space. For in so doing we are making the patient much more comfortable and lengthening the life of the teeth by preventing a pyorrhea that would be sure to result from a continuance of such conditions.

And whenever faulty fillings present that are food catchers and are giving the patient discomfort always take them out and correct the condition, for a filling, be it never so good from the standpoint of impermeability, is a failure if it allow the food to crowd between the teeth, and I have no hesitancy in advising the removal of such fillings.

I could give case after case of the loss of teeth from no other cause than that they had been improperly filled, in that the contact had been made so that the food crowded between the teeth and set up an irritation that in the end caused the loss of the teeth by reason of a superinduced pyorrhea. Operations so made are little less than malpractice, for they not only fail to properly correct the condition they were supposed to correct, but have made it worse than it was at the beginning.

In building up the contact with gold foil it is necessary to thoroughly condense the point that is to bear the brunt of contact, otherwise the constant abrasive effect of the movement of the teeth due to mastication will in time wear the contact points to planes.

We know that annealed gold is in its softest possible state, and that it is tempered, or hardened, by the process of beating or malleting, so that when we mallet a filling we are not only introducing the gold into the cavity and bringing the successive pellets into the closest possible union, thereby eliminating air spaces and increasing density, but we are also tempering the gold, and the more we beat it the more we harden it. So while it is of great importance to make the entire filling as hard as we can, it is doubly important to make the contact point hard, for it is this part of the filling that is to conserve the health of the septal tissue, and the more we study the cause of pyorrhea the more are we impressed with the necessity of preserving in its integrity the contents of the interproximal space.

In the restoration of the contact point with the gold inlay the same caution will apply. If the gold is cast in the pure state we will have an inlay that is altogether unreliable if we do not reinforce the contact point with a gold of greater resistance. I do not wish to enter into a discussion of the merits of the different karats of gold for the inlay at this time, and the present subject does not call for it; however, let me say that gold that is to bear the brunt of contact should never be pure gold, for we know that cast gold is in its softest condition, and it is not possible to temper the contact point of an inlay by the process of malleting, so the best thing to do is to sweat on a piece of 20 karat gold solder and let the contact come upon that. If the inlay is made of 22 karat plate or gold alloyed with 5 per cent. of platinum, the contact made by these metals will in most cases prove sufficiently hard to resist the forces of attrition for many years.

In the making of an amalgam filling the same care should be exercised in producing the contact as in a filling or inlay of gold. Here it is necessary, of course, to use a matrix, and this should be of the thinnest material consistent with the necessary strength. Either a previous separation or one obtained at the time of filling is necessary, for if not obtained the entire mesio-distal diameter of the tooth is not only not restored, but in the removal of the matrix the thickness of the metal will be taken away from the contact surface, and if the teeth do not move forward and fill that space a food pocket is left for the patient to remember you by every time he goes to the table. Adjust your matrix and then with your greatest force pack the amalgam into the cavity, especially against the contact point. The mallet, with considerable energy back of it, is most desirable. In this way considerable space can be gained, enough often to offset the loss suffered by the removal of the matrix.

I know of no operation that calls out the ecomiums of the patient or that ministers more to his comfort than the restoration of a contact that has been lost. In operating for a new patient some time ago I called attention to the fact that the fillings he had were lacking in contact and thus became food catchers, when he in surprise asked me if it were possible to fill teeth in such a way that the food did not always catch between them afterwards. I assured him that a filling made in any other way was worse than a failure, and have had the pleasure of showing him by making over his other fillings. And his is not an isolated case. If you want your patients to bless you, properly restore their contacts; but if you want them to curse you, neglect to do so, and you will have your reward.—*Dental Brief.*

